# A Singular Pet

**Published:** 1888-05

**Authors:** 


### A SINGULAR PET.

### DE GUSTIBUS NON EST DISPUTANDUM.

The *Omaha Herald* is responsible for the following story avouched by Judd Webb of Fremont. It is one of peculiar devotion—the only case of the kind ever known. The devotion is that of a boa constrictor thirty feet long, who seemingly has reasoning powers, understands language, sleeps on the hearth rug of a home, keeps the house free from rats and mice, and follows its master about the house like a dog. The huge serpent is to-day the property of the Rev. Goram Nail, now a resident of David county, North Carolina, and some twenty years ago a missionary in Africa.

"It seems that shortly before leaving Africa, he went into his house one morning, and, stepping into the kitchen, was horrified at finding his little child fondling on the floor with a young serpent. The two were caressing each other like lovers. There seemed to be no harm in it, and he kept the serpent in his family, and it soon became domesticated. It grew rapidly, and to-day it is thirty feet in length and has the power to crush an ox, or perhaps, an elephant. Since it was taken into the family it has never shown a savage disposition in any way. In 1870 the Rev. Dr. Nail returned to this country with his family, and brought the large serpent with him. The boa has been fed on milk, gruel and rabbits, but does not have the ravenous appetite that appearances would suggest. As it grew older it became more domesticated, and to-day it is the slave and pet of the child who has grown to be a woman.

A peculiar instance of its devotion is told. A few years ago its mistress had been away from home on a three weeks' visit. When she returned to the house the serpent recognized her at once, and, crawling to her shoulders, he rested his ugly head on her back. She had hard work to dislodge it, and it seemed that he feared that she would leave the house again. This is the longest time that the two had been separated. A few years ago the parents of the young lady died, and last year she was married, one of the stipulations of the union being that she should not be separated from her huge pet. Frank Buckland of London, in writing of this case, states that it is the only one known—an affection between a human being and a serpent.—*Omaha Herald.*

